# Time to Death and Its Predictor Among Outpatients With COPD: A Retrospective Cohort Study in Northwestern Ethiopia

**DOI:** 10.1002/hsr2.70896

**Published:** 2025-07-27

**Authors:** Yoseph Kassa, Anteneh Asmare Godana, Habtamu Geremew, Chalachew Gashu

**Affiliations:** ^1^ Department of Statistics, College of Natural and Computational Science Oda Bultum University Chiro Ethiopia; ^2^ Department of Statistics, College of Natural and Computational Science University of Gondar Gondar Ethiopia; ^3^ Department of Nursing, College of Health Science Oda Bultum University Chiro Ethiopia

**Keywords:** Cox‐PH model, Kaplan‒Meier, outpatients with COPD, survival data, time to death

## Abstract

**Background and Aims:**

Globally, chronic obstructive pulmonary disease (COPD) is one of the main causes of illness and death. Understanding the problem with respect to these factors is essential to finding a solution. Therefore, this study aimed to determine time to death and its predictors among outpatients with chronic obstructive pulmonary disease followed up in northwestern Ethiopia.

**Methods:**

A retrospective cohort study design was applied to collect relevant information on time to death from the medical patient's charts of 248 outpatients with COPD under follow‐up at the UGRH. The information was explored using log‐rank tests and Kaplan‒Meier plots. We used the Cox PH model for the time to death of outpatients with COPD.

**Results:**

Out of 248 COPD patients treated at UoG from January 1, 2020 to December 30, 2022, 82 (33.1%) patients died. The study population consisted of 248 patients who received COPD drug treatment during follow‐up, of whom 148 (59.7%) were male, and 145 (58.5%) resided in urban areas.

**Conclusion:**

The Cox proportional hazards (PH) model identified sex, occupation, comorbidities, age, HIV status, weight, marital status, and smoking as risk factors for time to death.

**Abbreviations**
AIC: Akaike's information criterion
BIC: Bayesian information criterion
CI: confidence interval
COPD: chronic obstructive pulmonary disease
HIV: human immunodeficiency virus
LRT: likelihood ratio test
REML: restricted maximum likelihood
UGRH: University of Gondar referral hospital
UoG: University of Gondar
WHO: World Health Organization

## 1 Introduction

The explanation of COPD has developed over the past several centuries. The most current definition of COPD is by nationwide and worldwide groups. Key words in each of these explanations include preventable, progressive, treatable, persistent airflow obstruction, inflammation, pulmonary, and exacerbation [1]. Over 59 million people have died, and 3,020,000 people have died from chronic obstructive pulmonary disease (COPD), accounting for approximately 5.1% of deaths worldwide, as described in the Global Burden of Disease 2004 updates [2].

There have been a few significant studies on the incidence of COPD in sub‐Saharan Africa. However, COPD has emerged as a growing public health challenge in the region due to widespread tobacco smoking and increased exposure to biomass fuels [3]. In many regions of sub‐Saharan Africa, 90% of agricultural households rely on biomass fuel for cooking and heating, exposing young children to acute lower respiratory infections and women to COPD. This widespread exposure significantly contributes to the high morbidity and mortality rates in the region [4, 5].

COPD is more common in older adults, and it is usually not diagnosed until an individual is at least 50 years old. Men are much more likely than women to develop COPD, and they also have a higher risk of dying from it.

COPD is more common in older adults, and it is usually not diagnosed until an individual is at least 50 years old. Men are much more likely than women to develop COPD, and they also have a higher risk of dying from it. The Scotland and North Britain regions have higher mortality rates than the South region, which is indicative of the fact that 20% of the population with the greatest population destruction has a prevalence that is almost twice as high [6].

The greatest collective problem of COPD is called an exacerbation or outbreak. Your physician may be devising a treatment plan to successfully manipulate flare‐ups. When left untreated, COPD can cause further serious health problems, together with cardiovascular ailment, breathing infections (colds, flu, and pneumonia), lung cancers, and depression [7].

Urbanization, industrial pollution, tanneries, and the indoor burning of biomass‐based fuels are all contributing factors to the increasing frequency of COPD, especially in Asian and African nations. In Africa, the prevalence of COPD is 13.4%, with a range of 9.4%–22.1%. On the other hand, reports of the illness in Asia vary from 3% to 22.2%, with an observed prevalence of 13.5%. Furthermore, COPD is a major contributor to hospital admission rates in many countries, which raises the expense of healthcare [8].

The incidence of COPD is increasing over time, primarily affecting developing nations such as Ethiopia. This suggests that more investigations are needed to detect and assess the causes or common practices of death in patients COPD patients.

Hence, the findings of this study are responsible for straightforward data concerning the factors that influence the time to death of patients with COPD, the expected time to death of outpatients with COPD, and the finest parametric survival models for the research of the COPD data set. The central objective of this study was to evaluate the survival time to death of outpatients with COPD at the UGRH, Gondar, Ethiopia.

This study is helpful for exploring the time to death in chronic obstructive pulmonary patients and identifying factors that contribute to COPD. The findings of this study provide the government and other relevant organizations with crucial information for developing policies, plans of action, and additional research to better understand the variables influencing chronic obstructive pulmonary outpatients.

## 2 Methods

### 2.1 Source Data

For this investigation, we considered a secondary data basis. The study design for this study was a retrospective cohort study. Patient charts, which cover the clinical and socio‐demographic records of all outpatients with COPD for the duration of continuation, were generated to extract time to death data from January 1, 2020 to December 30, 2022 to identify factors that affect chronic obstructive pulmonary disease. The COPD outpatients in this study were those who adhered to clinical therapy all the way up to hospital discharge, were transferred to another hospital, and/or had a combination of these situations. Patients can additionally end their treatment early or die before their treatment is considered as censored.

### 2.2 Eligibility Criteria

Patients who started COPD medication at the UGRH between January 1, 2020 to December 30, 2022 and who visited the branch clinic for prescription refills at least two times in their followed‐up were included in this study, whereas those who had less than two followed‐up visits and patients who were out of the study period were excluded.

### 2.3 Ethical Consideration

To obtain patient information for the purpose of this study, ethical approval was received from the College of Natural and Computational Science Ethical Approval Review Committee at the UoG (RN: 09/04/980/11/2022). With respect to confidentiality, all the data were kept confidential, and personal identification was not included.

### 2.4 Variables Under Study

#### 2.4.1 Dependent Variables

For this study, the time to death of COPD outpatients served as the response variable.

#### 2.4.2 Independent Variables

The explanatory variables that were risk factors for the change in time to death among outpatients with chronic obstructive pulmonary disease were sex, marital status, HIV status, age, residence, comorbidities (TB, diabetes, and asthma), weight, smoking status, education, and occupation.

### 2.5 Methods of Data Analysis

In this investigation, a survival model was used to govern the factors associated with survival time to death in outpatients with chronic obstructive pulmonary disease. The data were entered into SPSS and analyzed R 4.4.2 version software.

### 2.6 Survival Data Analysis

It involves the modeling and analysis of data, with the time up to an event occurring as the primary endpoint (time‐to‐death data). Survival statistics, also known as time‐to‐event data, track the passing of time [9].

### 2.7 Kaplan‒Meier Estimation

The Kaplan‒Meier estimator is a non‐parameter estimator of survival analysis that is used to designate the survivorship of patients both in detail and statistically [10]. The Kaplan‒Meier estimator, or product limit estimator, is the estimator analyzed by most software; it comprises information for censored and event observations. The Kaplan‒Meier estimator plot of the survival function is a step function that decreases with each passing time [11].

### 2.8 Model Selection

It is vital to examine several models using different methods and techniques to choose the parsimonious model that best fits the available data. Therefore, a crucial component of statistical inference is the comparison of several models [12]. The most frequently used model comparison approaches are the likelihood ratio test (LRT) approach, the Bayesian information criterion (BIC), and Akaike's information criterion (AIC).

## 3 Results

The results of the statistical analysis and discussion are presented in this section. The survival time to death among outpatients with chronic obstructive pulmonary disease was fitted to the research objectives.

The survival endpoint of interest was the time to death among outpatients with COPD. Censored patients were those patients who were unable to follow up or transfer to other hospitals or those who did not die until the end of the study period. Hence, the time‐to‐event was 3 months. Thus, of the 248 patients with COPD treated at the University of Gondar from January 2020 to December 2022, 166 (66.9%) were censored, whereas the remaining 82 (33.1%) patients died.

A total of 248 patients who received COPD drug handling during follow‐up were included, of whom 100 (40.3%) were females and 103 (41.5%) lived in rural areas. Approximately 116 (46.8%) had comorbid diseases, and 25 (10.1%) COPD patients had HIV. Approximately 78 (31.5%) of the participants were smokers with COPD, and with respect to marital status, 64 (25.8%), 14 (5.6%), 140 (56.5%), and 30 (12.1%) were single, divorced, married, and widowed, respectively. With respect to educational status, 120 (48.4%), 16 (6.5%), 14 (5.6%), and 98 (39.5%) patients were diploma & above, secondary educated, primary educated, and not educated, respectively. Nearly 82 (33.1%), 58 (23.4%), 68 (27.4%), and 40 (16.1%) patients were merchants, other occupations, farmers, and government workers, respectively, as presented in Table 1.

**Table 1:** Independent variable summary statistics.

Variables	Explanatory variable categories	*n* (%)	Censored	Death
Residence	Rural	103 (41.5%)	86	30
	Urban	145 (58.5%)	80	52
Sex	Male	148 (59.7%)	77	58
	Female	100 (40.3%)	89	24
Marital‐status	Single	64 (25.8%)	34	26
	Married	140 (56.5%)	88	42
	Widowed	30 (12.1%)	34	6
	Divorce	14 (5.6%)	10	8
Smoking	Non smoker	170 (68.5%)	138	40
	Smoker	78 (31.5%)	28	42
Occupation	Farmer	68 (27.4%)	56	20
	Merchant	82 (33.1%)	50	32
	Government employed	40 (16.1%)	24	16
	Others	58 (23.4%)	46	22
Education	No educated	98 (39.5%)	80	20
	Primary educated	120 (48.4%)	68	4 8
	Secondary educated	14 (5.6%)	10	6
	Diploma and above	16 (6.5%)	8	8
Comorbidities	No	132 (53.2)	106	34
	Yes	116 (46.8)	50	48
HIV	No	241 (89.9%)	188	65
	Yes	25 (10.1%)	8	17
	Description of continuous variables			
	Min	Max	Mean	SD dev.
Baseline age	20	82	45.01	13.69
Baseline weight	43	70	56.07	6.99

### 3.1 Nonparametric Survival Data Analysis

#### 3.1.1 Kaplan‒Meier Survival Curves

The first indications of the form of the survival function are given by the Kaplan‒Meier survival curves for every research variable. K‒M survival curves showing whether there was variation in the time to death among COPD patients with different categories of covariates.

**Figure 1:**
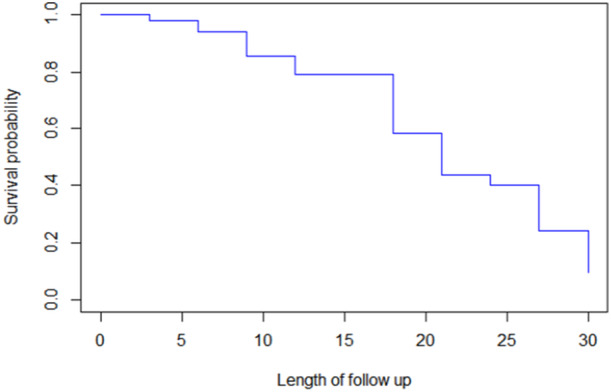
Overall estimate of the Kaplan‒Meier survival function plot of outpatients with COPD.

Figure 1 shows that the overall survival probability of outpatients with COPD did decrease with increasing stage, as did the corresponding increase in survival time.

### 3.2 Exploring the Time to Death of Outpatients With COPD With Categorical Variables

In this study, Kaplan‒Meier survival curves were used to examine whether there was a difference in the time to death among COPD patients in different categories of predictors.

**Figure 2:**
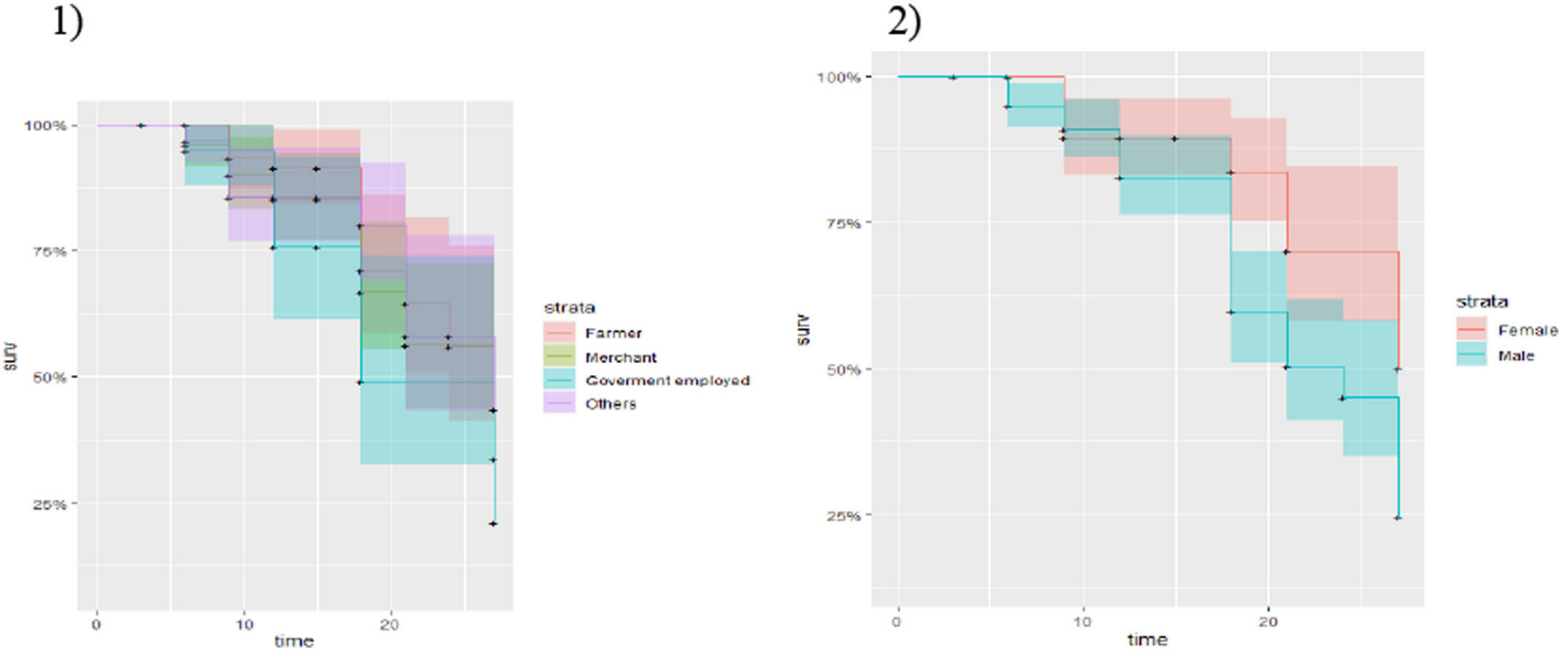
Time to death by categorical factors for COPD outpatients.

Figure 2 displays the time to death of patients with COPD according to specific factors in this investigation. However, the results revealed that (1) no significant difference in the time to death among occupational patients with COPD was observed, with a corresponding increase in survival time. (2) Female COPD patients died more quickly than male COPD patients did, and this result was attributed to the lower survival probability of male patients.

### 3.3 Log‐Rank Test for Each Categorical Variable in the Study

The log‐rank test was used to check for important differences in survival among groups of factors. The null hypothesis was that there would be no major difference in the survival records of several sets of categorical variables, according to the null hypothesis.

The results in Table 2 show that education, sex, smoking status, comorbidities, marital status, and HIV status of patients with COPD were significantly different in terms of survival between these patients (time to death). However, there was no indication of statistically significant variation in the patients' overall survival experiences according to their occupation of work and place of residence.

**Table 2:** Log‐rank test for each categorical variable.

Variable	*χ* ^2^	DF	*p* value
Residence	4.1	1	0.09
Comorbidities	10.8	1	**< 0.0001**
Marital status	22.9	3	**0.004**
Sex	11.5	1	**< 0.0001**
Occupation	2.8	3	0.4
HIV	30.4	1	**< 0.0001**
Comorbidities	10.8	1	**< 0.0001**
Education	15.4	3	**< 0.0001**

*Note:* Bold values indicate statistically significant differences.

### 3.4 Multivariable Analysis of the Cox Proportional Hazard Model

In this study, multivariable analysis of the Cox proportional hazards model was accomplished by using all the significant predictors at the 25% significance level in the univariable analysis.

The results from Table 3 revealed that patient sex, comorbidities, smoking status, HIV status, age, occupation, marital status, and weight were significantly related explanatory variables for time to death. On the other hand, educational status and patient residence were not statistically important predictors of the time to death among COPD outpatients at the 5% level of significance.

**Table 3:** Results of the final Cox PH model for the time to death of COPD patients.

Variable	Categories	Estimate	S.E	HR	*p* value
Weight	—	0.34309	0.56893	1.410	< 0.001
Residence (ref = rural)	Urban	−0.25453	0.35029	0.775	0.8
Occupation (ref = farmer)	Government employed	1.63343	0.33577	5.121	< 0.001
	Merchant	1.63343	0.33577	5.121	< 0.001
	Other	1.56438	0.46775	4.780	< 0.001
Sex (ref = male)	Female	1.03680	0.36827	2.820	< 0.001
Smoking (ref = nonsmoker)	Smoker	1.42164	0.67934	4.144	< 0.001
Age	—	−0.67489	0.26073	0.509	< 0.02
HIV (ref = no)	Yes	1.55493	0.27325	4.735	< 0.001
Education (ref = no‐educated)	Primary education	0.64265	0.40028	1.902	0.22
	Secondary education	0.64265	0.40028	1.902	0.22
	Diploma and above	0.31485	0.38281	1.370	0.54
Marital status (ref = single)	Married	1.17350	0.33644	2.233	0.003
	Divorce	1.48067	0.27931	4.396	0.019
	Widowed	0.72013	0.01939	2.055	0.15
Comorbidities (ref = no)	Yes	0.71094	0.01140	2.036	< 0.001

Abbreviations: ref = reference category for categorical predictors, SE = standard error.

### 3.5 Survival Model Diagnostics

To verify the Cox PH model of the survival model's proportional hazards assumption, the Schoenfeld residuals and the official statistical test were used. The Cox proportional hazard assumption for the survival model of the time to death for COPD patients was checked graphically via Schoenfeld residuals.

**Figure 3:**
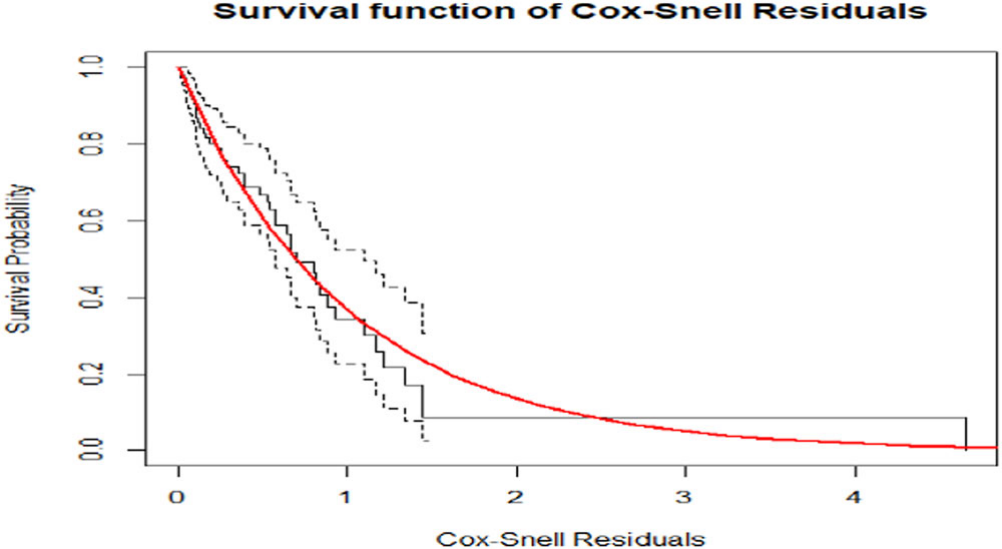
Cox–Snell residual plots of the survival model of COPD patients.

Figure 3 shows the Kaplan‒Meier estimate of the Cox–Snell residuals along the red line, along with the model diagnosis based on the Cox–Snell residuals and 95% CIs. This result suggested that the survival progression model explained the data well.

### 3.6 Interpretation of Results

Table 3 displays the results for time to death among outpatients with COPD. According to the Cox‐PH model results, interpretations of the parameters of the survival model were made.

The estimated hazard ratio of death for patients of married marital status was e^1.1735^ = 2.233, implying that for a married marital status, death is 2.233 times more likely to occur than for a single marital status among outpatients with COPD, with other variables held constant. The estimated hazard ratio of death for patients by sex was e^1.0368^ = 2.82, implying that for female patients, death is 2.82 times more likely to occur than for male COPD outpatients, with additional variables held constant. According to the Cox‐PH model, the predictable hazard ratio of death for variable weight was e^0.0368^ = 1.04, inferring that for a one‐element increase in patient weight, the hazard of death among COPD outpatients meaningfully increased by 41% when additional variables were held constant.

The estimated hazard ratio of death for patients in merchant occupations was e^1.63343^ = 5.121, implying that, holding other variables constant, the risk of death for merchants was 5.121 times higher than that of farmers among outpatients with COPD. In addition, the estimated hazard ratio of death for patients who were tasked in other fields of work was e^1.56438^ = 4.78, implying that for other fields of occupation, death is 4.78 times more likely to occur than farmer death among outpatients with COPD, with additional variables held constant. The estimated hazard ratio of deaths due to related disease was e^0.71094^ = 2.036, implying that the existence of related disease of death is 2.036 times more likely to occur than the nonappearance of related disease among COPD outpatients, with additional variables held constant. Additionally, the estimated hazard ratio of death for patients with HIV infection was e^1.55493^ = 4.735, implying that for patients with HIV infection, death is 4.735 times more likely to occur than the nonexistence of HIV among COPD outpatients, with additional variables held constant. The estimated hazard ratio of death for patients who had a habit of smoking was e^1.42164^ = 4.144, implying that patients who had a habit of smoking were 4.144 times more likely to have a habit of smoking than nonsmokers among COPD outpatients, with additional variables held constant.

## 4 Discussion

In this study, Cox‐PH models were fitted. Cox proportional hazards model for time to death in chronic obstructive pulmonary outpatients. For the analysis of survival data, nonparametric survival analysis techniques (Kaplan‒Meier and log‐rank tests) and Cox proportional hazard regression analysis were applied to investigate the factors determining the time to death in COPD outpatients.

The predictable hazard ratio of death for patients who have HIV infection was e^1.55493^ = 4.735, implying that for patients with HIV infection, death is 4.735 times more likely to occur than for patients without HIV infection among COPD outpatients, with all variables held constant. This study aligns with the findings of studies conducted by Pefura‐Yone et al., among patients with HIV poison in this situation, and who, for many patients, have experienced pulmonary tuberculosis. The existence of chronic respiratory symptoms and other factors identified in this study should trigger specific investigations to determine the probable fundamentals of COPD. Such a proactive approach helps optimize the care of these patients [13].

The number of outpatients with COPD who had related diseases was significantly higher by 2.036 times than that of COPD patients who had no related diseases. This finding is consistent with those of other studies by Figueira‐Gonçalves et al. Arterial hypertension (59.5%), dyslipidemia (54.3%), and type II diabetes (31.2%) were the most often reported associated morbidities; heart disease affected 32% of the patients. Patients referred for chronic obstructive pulmonary disease to Canary Island Pneumology outpatient clinics had high rates of heart disease, type II diabetes mellitus, and active smoking [14].

A study revealed that males were 2.820 times more likely than females were, which means that males had a 2.82 times greater probability of death than females were. Patient sex was also determined to be significantly associated with time to death. This result contradicts the findings of Yang et al., who reported no significant association between patient sex and time to death [15].

The weight of the patient was shown to have a statistically significant influence on the time to death. By holding the other variables constant, this means that there was a 41% increase in the predicted hazard for every element increase in weight. The hazard ratio for smoking status was HR = 4.144. Compared with nonsmokers, smokers are at greater risk of developing COPD when their risk is compared with that of nonsmokers. Compared with nonsmokers, smokers are at greater risk of developing COPD when their risk is compared with that of nonsmokers. Compared with nonsmokers, smokers are at greater risk of developing COPD. This finding is consistent with a study by Thapa et al., which revealed that COPD patients who were smokers were 4.144 times more likely to die than nonsmokers [16].

The limitation of this study is the insufficient data concerning risk factors for COPD, such as COPD stage, COPD treatment, biomass fuels, body mass index, and number of exacerbations. Therefore, we advise that investigators add these factors to their findings. Our results reflected the situation in a small number of participants and did not represent the whole nation state. Finally, in this study, the time to death could not be used to determine repeated measures of possible risk factors.

## 5 Conclusion

The vital goal of this study was to identify the potential hazard issues affecting the time to death of chronic obstructive pulmonary outpatients. This retrospective cohort study included 248 patients with COPD who were receiving follow‐up care for outpatients with COPD at UGOH, Gondar, Ethiopia, between January 1, 2020 and December 30, 2022.

According to the Cox‐PH survival analysis, the marital status of patients, sex, smoking status, comorbidities, weight, HIV status, and occupation of patients were statistically important factors influencing the time to death among patients with COPD.

On the basis of these findings, we recommend the following idea: patients who have comorbidities or diseases, that is, diabetes, TB, and asthma, are at greater risk of default than are those who have no comorbid diseases, that is, diabetes, TB, and asthma. Thus, in hospitals, special attention should be given to patients who have the above comorbid diseases. Continuous, timely medical care should be important for patients to attain or improve their lives in a sustainable way.

## Author Contributions

**Yoseph Kassa:** investigation, conceptualization, writing – original draft, methodology, validation, software, data curation. **Anteneh Asmare Godana:** validation, visualization, formal analysis, software, supervision, methodology. **Habtamu Geremew:** visualization, validation, investigation, resources, formal analysis, project administration. **Chalachew Gashu:** software, visualization, validation, methodology, conceptualization, resources.

## Ethics Statement

The University of Gondar College of Natural and Computational Science Ethical Clearance Review Committee granted ethical permission (reference number: 09/04/980/11/2022), waiving the need for informed consent to gather and retrieve patient medical chart data. With respect to privacy, no personal information was linked to any particular person, and all the data was kept secret. The enrollment of the study's human subjects was performed in accordance with the Declaration of Helsinki.

## Consent

The authors have nothing to report.

## Conflicts of Interest

The authors declare no conflicts of interest.

## Transparency Statement

The lead author Yoseph Kassa affirms that this manuscript is an honest, accurate, and transparent account of the study being reported; that no important aspects of the study have been omitted; and that any discrepancies from the study as planned (and, if relevant, registered) have been explained.

## Data Availability

Data access may only be obtained by contacting the corresponding author; data cannot be placed in open‐access repositories for ethical reasons.
